# Epidemiology, Transmission, and Evolution of Japanese Encephalitis Virus

**DOI:** 10.3390/microorganisms13061226

**Published:** 2025-05-27

**Authors:** Chengcheng Peng, Huiling Qin, Fan Yu, Yujia Hao, Yuge Yuan, Wenzhou Ma, Duo Zhang, Pengpeng Xiao, Nan Li

**Affiliations:** Wenzhou Key Laboratory for Virology and Immunology, Institute of Virology, Wenzhou University, Wenzhou 325035, China; 18741083225@163.com (C.P.); qinhl130@163.com (H.Q.); yufan0009@163.com (F.Y.); unih4519@163.com (Y.H.); yuge202210@163.com (Y.Y.); ma1172864201@outlook.com (W.M.); zd1251071348@163.com (D.Z.)

**Keywords:** Japanese encephalitis virus, whole-genome analysis, phylogenetics, phylogeography, virus evolution

## Abstract

The Japanese encephalitis virus is an arbovirus that causes severe damage to the central nervous system. At present, there are still 67,900 cases of Japanese encephalitis worldwide every year, which poses a global public health concern and causes great economic losses to animal husbandry. In this study, we analyzed the epidemiology, transmission, and evolution of JEV based on the NCBI database. E and NS1 were emphatically analyzed for amino acid variation and predicted protein structure. Gene recombination and the evolutionary rate of JEV were analyzed using RDP 4 and BEAST. The maximum clade credibility tree of E was reconstructed to estimate the time of the most recent common ancestor. Chinese genotype Ⅰ (GI) strain recombination events occurred in the C, M/PrM, E, NS2A, NS4B, and NS5 proteins, and genotype III (GIII) strains occurred in the E, NS1, NS3, NS4A, and NS5 proteins. The average evolutionary rates of JEV were comparable (3.3830 × 10^−4^, 2.0481 × 10^−4^, 3.5650 × 10^−4^, 2.2423 × 10^−4^, 3.0844 × 10^−4^, and 1.9757 × 10^−4^ substitutions/site/year for the JEV-I whole genome, JEV-III whole genome, JEV-I E gene, JEV-III E gene, JEV-I NS1 gene, and JEV-III NS1 gene, respectively). The MCC tree revealed the evolutionary order was GⅢ, GⅠ, GⅤ, GⅡ, and GⅣ. This study was expected to provide theoretical support for vaccine development and comprehensive prevention and treatment of JEV.

## 1. Introduction

Japanese encephalitis (JE) is a zoonotic infectious disease caused by the Japanese encephalitis virus (JEV), which invades the central nervous system. China is a high-incidence area of JE, and the average incidence of JE accounts for more than 80% of the global annual incidence. The case fatality rate can be as high as 30%. Most patients started with fever and the main manifestations of central nervous system damage symptoms, including severe headache, nausea and vomiting, and mental behavior abnormalities that severely affected the quality of life of patients [1]. When infected, JEV causes great economic losses to the pig industry, such as reproductive disorders in piglets, breeding pigs, and sows. It can also cause pregnant sow abortion, stillbirth, and acute inflammation of boars [2,3]. For example, Australia suffered from a JEV outbreak in 2021 and mid-2022. JEV was detected in about 80 pig farms, affecting about 60% of the Australian pig industry. Losses were estimated at USD 215,000 to USD 250,000 per 1000 sows [4].

JEV belongs to the genus *Orthoflavivirus*, family *Flaviviridae*. The nucleic acid type is a single positive-stranded RNA, about 11 kb long, encoding three structural proteins and seven nonstructural proteins. The structural proteins are C, PrM/M, and E. Nonstructural proteins include the NS1, NS2a, NS2b, NS3, NS4a, NS4b, and NS5 proteins [5]. Structural protein E is a critical antigen protein that can recognize cell surface receptors, mediate receptor binding, and affect membrane fusion [6]. The nonstructural protein NS1 is a multifunctional protein involved in viral replication, assembly, and immune evasion [7]. JEV exists in five distinct genotypes, each of which can be distinguished based on the nucleotide sequence of the E protein gene [8,9]. At present, the main prevalent strains of JEV are genotype Ⅰ (GI) and genotype Ⅲ (GIII) in Asia, while GI is gradually replacing GⅢ as the dominant genotype [10].

Mosquitoes are the main vector of JEV. JEV has been isolated from 30 species of mosquitoes [11,12]. The main vectors of JEV include *Culex*, *Armigeres*, and *Aedes* [13]. Among these, *Culex tritaeniorhynchus* Giles is predominant in the Afrotropical, Oriental, and Palearctic Region; *Armigeres subalbatus* is found in the Indo-Oriental and Palearctic Region; and Anopheles sinensis occurs in both the Oriental and Palearctic Region as a key mosquito species. Apart from the mosquitoes, Culicoides or bats can also act as vectors for JEV [14,15]. And mosquitoes can transmit JEV to humans through the pig–mosquito–human transmission cycle. Adult mosquitoes can carry the virus through the winter and become the storage host of JEV [16,17]. Pigs are the main source of infection of JEV, and JE spreads rapidly in nature through the pig–mosquito–pig secondary transmission cycle [8,18]. For pigs, CD4 has been proven as a receptor in PK-15 cells [19]. The fraktalkine receptor CX3CR1 is also increased in human microglia [20]. JEV has tissue tropism for neural and reproductive tissues in both pigs and humans [21,22]. In addition, studies have shown that HSPG, HSP70, HSP90B, vimentin, laminin receptor, and α5β3 integrin have also been presented as cell receptors for JEV [23].

The existing JEV vaccines are mainly based on genotype III. Consequently, the antigenic differences among JEV genotypes may impact the protective efficacy of existing GIII vaccines [24]. Although studies have shown that the current JEV vaccine has a good cross-protective capacity for GI-GIV, GIII vaccines have a weakened ability to neutralize the GV strains [25,26]. In addition, as the JEV evolves and mutates, the protective efficacy of vaccines may be weakened. Therefore, understanding the genetic and antigenic characteristics of different genotypes is essential for advancing the research and development of genotype-specific vaccines. In this study, we compiled amino acid sequences encoding the E and NS1 genes, highlighting mutations that had a high number of sequences. Additionally, we investigated glycosylation and palmitoylation across all genotypes. Further experiments could be conducted to examine the impact of amino acid variants and specialized functional sites on viral virulence or other functions.

JEV is dynamically evolving, and its virulence and immunogenicity change in response to unusually dynamic factors. Therefore, monitoring the genomic evolutionary dynamics of JEV is essential to gain insight into the epidemiology and integrated control strategies [27,28]. However, much of the current research on JEV is limited to outbreaks within particular regions or over limited timeframes. This study aims to provide a comprehensive characterization of the epidemiology, transmission, genomics, and evolution of JEV based on the whole-genome sequences of JEV in the NCBI before the start of this study. Compared with existing studies, this study encompasses a broader temporal scope and a wider geographical range. By using bioinformatics tools, we further investigated the global distribution, molecular structure, gene recombination, and evolutionary processes of JEV based on the whole-genome sequence dataset. The source, genotype, and uploaded condition were clarified. By mapping the global distribution of hosts and the main vector of JEV, *Culex tritaeniorhynchus,* basic data were provided for the prevention and control of JEV. A comprehensive analysis of the JEV genetic evolution was further analyzed, and the mutation sites of the JEV E and NS1 proteins, and the protein structure and function sites were predicted to provide the basis for JEV vaccine research and development. We constructed a phylogenetic tree to analyze the relationship between different JEV strains and the maximum clade credibility (MCC) tree to trace their origins. This work offers valuable insights for epidemic monitoring and early warning systems related to JEV outbreaks. The paper aimed to gain a deeper understanding of JEV through molecular biology and bioinformatics analyses, which will provide a basis for future vaccine development, evolutionary tracking, and comprehensive prevention.

## 2. Materials and Methods

### 2.1. Global Distribution of JEV

A total of 303 whole-genome sequences of JEV uploaded before October 2023 from the NCBI database were downloaded. The details of the collection date, origin, genotype, and host were collected. Of these, 295 had country information, and 161 JEV sequences had genotype information. According to the source statistics of the whole sequences of JEV, the MapChart website (https://www.mapchart.net/world.html accessed on 5 May 2024) was used to map the global and Asian distribution of JEV. The distribution of JEV in various provinces of China was mapped according to the sequence genotype information.

### 2.2. Host Diversity of JEV

Host information of the downloaded JEV whole-genome sequence was collected and mapped using Excel according to the collection date and host species, respectively. MapChart was used to make the global distribution map of JEV hosts, while Excel was used to count the number of JEV hosts. The Biorender website (https://www.biorender.com/ accessed on 7 April 2024) and PPT were used to add animal contours.

### 2.3. Vector Distribution and Circulation of JEV

The global distribution of *Culex tritaeniorhynchus*, the main vector of JEV, was obtained from the GBIF website (https://www.gbif.org/zh/ accessed on 16 November 2023). According to reports in the literature on the JEV transmission cycle and host, the Biorender website was used to map the JEV transmission cycle.

### 2.4. Gene Variation and Protein Structure Prediction of JEV E and NS1

The sequences of GI and GIII were selected from all the downloaded JEV genome sequences. The E and NS1 genes were intercepted by MEGA X [29] software v10.2.6 after comparison and were translated into amino acids. The functional variable sites were used to quickly screen the different amino acid sites, and the amino acid variation was calculated. From different amino acid sequences of GI and GIII, a sequence with no mutation site was selected, JEV E GI was JN381841.1, and JEV E GIII was KF907505.1. The JEV NS1 GI was JN381845.1, and the JEV NS1 GIII was KX945367.1. The amino acid sequence was input into the SWISS-MODEL [30,31,32,33,34] website (https://swissmodel.expasy.org/ accessed on 3 February 2024) and the protein prediction model was derived, respectively [35]. The NS1 amino acid sequence of the model predicted by the SWISS-Model was incomplete, so I-TASSER [36,37,38] (https://zhanggroup.org/I-TASSER/ accessed on 11 March 2024) was used to further predict the complete model [39]. Then, the protein model was modified in PYMOL v2.6.0a0 [40], and finally, the position of the amino acid mutation was marked in the protein structure map by Wizard-Mutagenesis-Protein [41].

The epitope information known to JEV was downloaded from the IEDB database (http://www.iedb.org/ accessed on 26 April 2025), limited to Response Frequency (RF) values greater than 0.25 [42]. The amino acid coding sequences used for modeling were compared using ImmunomeBrowser to obtain specific epitope information, which was visualized using PYMOL v2.6.0a0.

### 2.5. Prediction of Functional Sites of JEV

Using MEGA X v10.2.6 software, the entire genome sequence of JEV was truncated to obtain separate nucleotide sequences of JEV C, PrM/M, E, NS1, NS2A, NS2B, NS3, NS4A, NS4B, and NS5. All of them were translated into amino acid sequences and imported into NetNGlyc 1.0 [43] (https://services.healthtech.dtu.dk/services/NetNGlyc-1.0/, accessed on 19 January 2024) and GPS-SUMO 2.0 [44] (https://sumo.biocuckoo.cn/online.php, accessed on 6 March 2024) online websites for predicting N-glycosylation and SUMOylation functional sites [45,46]. Afterward, CSS-Palm 2.0 software was used to predict the palmitoylation functional sites of various proteins in JEV [47,48].

### 2.6. Phylogenetic Analysis of JEV

All the JEV whole-genome sequences downloaded from NCBI were screened, and 159 with detailed source, genotype, and host information were selected. After comparison using MEGA X 10.2.6 software, the command “FastTree -gtr -nt BiDuiJieGuoFASTA. FAS>tree_file” was entered in FastTree 2.1.11 software [49], and the phylogenetic tree using the GTR+CAT model was established [50,51]. The iTOL online site (https://itol.embl.de/ accessed on 13 May 2025) was used to beautify the phylogenetic trees [52].

### 2.7. Analysis of Gene Recombination and the Evolutionary Rate of JEV

The whole-genome sequences of GI and GIII from China were selected for a specific analysis of recombination events. The sequences were uploaded to the online version of MAFFT (https://mafft.cbrc.jp/alignment/server/index.html accessed on 2 April 2024) and the files were analyzed for gene recombination using RDP 4 [53]. Seven kinds of methods other than LARD were selected for the analysis, and events detected by three or more methods and the *p*-value cutoff of 0.05 were considered true recombination events.

Recombinant sequences were removed from all the JEV whole-genome sequences with information on time, country, genotype, and isolation location using RDP 4. GI and GIII sequence alignments were performed, respectively, using the online version of MAFFT (https://mafft.cbrc.jp/alignment/server/index.html accessed on 2 April 2024) and then the best-fit model of maximum likelihood trees was identified using IQ-TREE v2.2.2.6 [54]. According to the Bayesian Information Criterion (BIC), the best-fit model for GI was GTR+F+R2, and for GIII, it was GTR+F+G4. And then, the maximum likelihood trees with the best-fit model were generated. The tree file was uploaded to TempEst v1.5.3 [55] to determine whether a temporal signal existed based on the correlation coefficient.

The nucleotide substitution models for the GI and GIII alignment sequences were identified, respectively, using ModelFinder v2.2.0 [56] in PhyloSuite v1.2.3 [57] based on BIC, which was suitable for BEAST v1.10.4 The best nucleotide substitution model was GTR+F+G4. Marginal likelihood estimation (MLE) using path sampling (PS)/stepping-stone sampling (SS) was performed to find the best combination of the clock model and tree prior. Therefore, the best combination of the clock model and tree prior was the Uncorrelated relaxed clock (UCLD) and Coalescent: GMRF Bayesian Skyride.

Based on the best nucleotide substitution model and a combination of the clock model and tree prior, a Bayesian analysis was performed, respectively, with the whole-genome sequences, E gene, and NS1 gene from the GI and GIII sequences using BEAST v1.10.4 [58] to obtain the substitution rates. Three sets of sequences were generated, respectively, for GI and GIII by selecting 80% sequences randomly. Each set of GI consisted of 37 sequences, while GIII consisted of 25 sequences. The substitution rate was reported for 3 runs, and the average substitution rate was calculated for the statistical analysis. Markov Chain Monte Carlo (MCMC) was run for 2 × 10^8^ generations for the whole-genome sequences and 10^8^ generations for the E gene and NS1 gene, and 10% was set as the burn-in value [59]. Tracer v1.7.2 [60] was used to probe the convergence of the chains according to the Effective Sampling Size (ESS). If the ESS < 200, two log files with the same parameters were combined using LogCombiner v1.10.4 to ensure an adequate ESS.

The Mixed-Effects Model of Evolution (MEME) [61] was used to monitor the selective pressure sites in the E and NS1 genes. Amino acid composition maps on episodic diversifying sites were plotted using the WebLogo online site (https://weblogo.berkeley.edu/logo.cgi accessed on 13 May 2025) [62]. Single-Likelihood Ancestor Counting (SLAC) [63], Fast Unconstrained Bayesian AppRoximation (FUBAR) [64], and Fixed-Effects Likelihood (FEL) [63] from Datamonkey [65,66,67,68,69] were used to further examine positive selection pressure sites. Sites detected by more than 2 algorithms were considered positively selected sites. The Branch-Site Unrestricted Statistical Test for Episodic Diversification (BUSTED) [70] was performed for a gene-wide positive selection test.

### 2.8. Construction of the MCC Tree for JEV E

The above 83 screened whole-genome sequences were compared by MEGA X, and the E gene was extracted and saved. ModelFinder software v2.2.0 was used to find the best alternative nucleotide model, and the best alternative nucleotide model was TNe+G4. BEAUTi software v1.10.4 set the parameters and used the previous combined model. In BEAUTi software Operators, the value of the ucld mean weight parameter was expanded tenfold. The length of the chain in MCMC was set to 200,000,000, and finally, the result XML file was run using BEAST. Tracer v1.7.2 software was used to check the convergence of the above analysis results. Generally, the ESS of all the parameters was greater than or equal to 100. Tree Annotator software v1.10.4 was used to set the parameters and generate the maximum clade credibility tree (MCC). Finally, Figtree v1.4.4 was used to edit and visualize the MCC tree [51,71,72].

## 3. Results

### 3.1. Global Distribution and Genotype Characteristics of JEV

**Figure 1:**
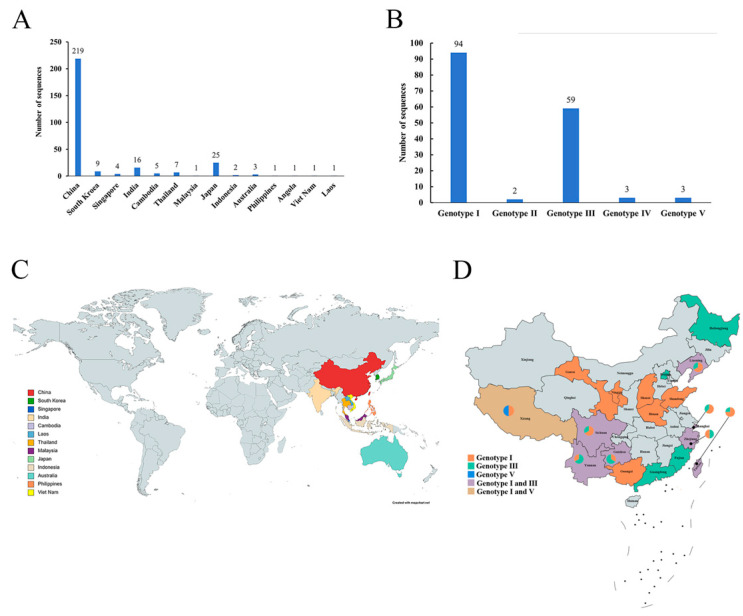
Isolation country and genotype of JEV. (**A**) Number of JEV whole-genome sequences across different countries. The horizontal coordinate was the country, and the vertical coordinate was the number of sequences. (**B**) Genotype distribution of JEV whole-genome sequences. The horizontal coordinate was the genotype, and the vertical coordinate was the number of sequences. (**C**) Global distribution of uploaded JEV whole-genome sequences. (**D**) Genotype distribution of JEV whole-genome sequence in China. The pie chart in the figure represents the proportion of different genotype sequences within this dataset.

Based on the downloaded sequence information, it was found that the JEV sequences came from 14 countries. The data indicated that the highest number of sequences came from China, with 219, followed by Japan (Figure 1A; Table S1). In terms of sequence genotype, genotype I (GI) and genotype III (GIII) were still the dominant genotypes. The GII sequences had only two sequences, respectively, from Australia and Indonesia, while the GIV sequences had only three sequences—two from Australia and one from Indonesia. GV had three sequences, which came from Singapore, China, and Malaysia (Figure 1B). It was worth noting that 99% of JEV sequences were uploaded by Asian countries; among these, 75% were contributed by China across 18 provinces. This suggested that JEV posed a threat of spreading in Asian countries, and China paid high attention to JE and researched it more. From the data uploaded globally, it can be concluded that GI has emerged as the new major genotype.

In China, three genotypes, I, III, and V, had emerged. However, the predominant strains were still GI and GIII. The JEV sequences of GI were widely distributed across 13 provinces in China, while GIII was mainly distributed in 10 provinces. In the statistics, Yunnan Province reported the highest number of uploaded sequences. It may be related to the climate and diversity of mosquito species in Yunnan Province. There were eight provinces where both genotypes coexisted, most of which were dominated by GI and GIII. Only Tibet exhibited occurrences of both GI and GV. The Tibetan province, however, was geographically distant from other countries where GV has been detected. JEV outbreaks might exist in some countries but have not been detected.

### 3.2. The Relationship of the JEV Upload Sequence to the Host and Vector

**Figure 2:**
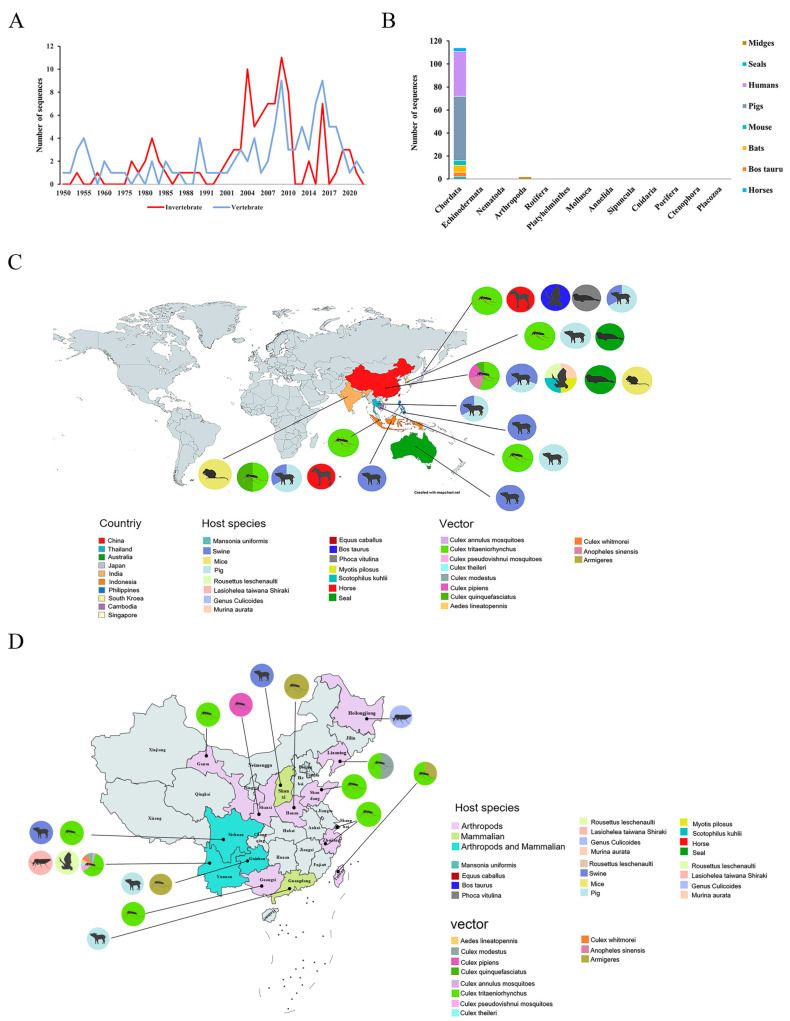
Diversity of hosts and vectors of JEV. (**A**) The number of sequences classified as vertebrates in the uploaded sequences over time. Blue fold was vertebrate; red was invertebrate. (**B**) The number of whole-genome sequence sources classified according to phylum, in which mosquitoes were removed as vectors. (**C**) Global distribution of hosts and vectors of JEV whole-genome sequences. (**D**) Distribution of hosts and vectors of JEV in China. Different colors represent different hosts and vectors, with the scale of the pie chart reflecting the proportion of sequences corresponding to each vector or host.

Host distribution and transmission cycles revealed that mosquito vectors were an important factor in JEV transmission. During the 20 years from 1990 to 2010, the number of JEV whole-genome sequences uploaded to the NCBI database increased significantly. The magnitude of variation fluctuations was comparable for both invertebrates and vertebrates. And the invertebrates in the statistical sequence sources were mainly mosquitoes, while the vertebrates were mainly humans and pigs. Furthermore, the timing of the peaks of both datasets was more consistent. Both invertebrates as vectors and vertebrates as hosts were important for JEV transmission (Figure 2A). According to the obtained host information, it was evident that all JEV hosts belong to the phyla Chordata and Arthropoda. JEV hosts within the Chordata phylum were diverse, including pigs, mice, horses, and bats. In contrast, only mosquitoes and midges belong to the arthropod phylum (Figure 2B). According to the statistics, mosquitoes served as the primary vectors for JEV, with a total of 12 species, especially *Culex tritaeniorhynchus*. Of these, 45.8% of the JEV whole-genome sequences were extracted from mosquitoes, and 25.7% were *Culex trituberculatus*. In addition, the JEV hosts also included two species of midges, four species of bats, domestic pigs, and wild boars (Figure 2C). Among these hosts, horses, pigs, and humans were the most susceptible chordate species. Most national JEV extraction sources were from pig and mosquito. Publicly available data from China’s National Bureau of Statistics showed that by the end of 2023, there were approximately 726 million pigs and around 3.6 million horses in China. Therefore, controlling outbreaks of JEV was important for public safety as well as the aquaculture industry. In China, JEV hosts and mosquito vectors were mainly distributed in 14 provinces. Among these regions, mosquitoes and pigs showed widespread distribution patterns. The concentrated breeding of JEV-susceptible animals may facilitate infection transmission while exacerbating treatment challenges due to extensive mosquito populations. Furthermore, animal migration played a significant role in affecting the spread of JEV. Therefore, focusing on the distribution of JEV vectors and susceptible hosts will be essential in enhancing strategies aimed at controlling future outbreaks (Figure 2D).

**Figure 3:**
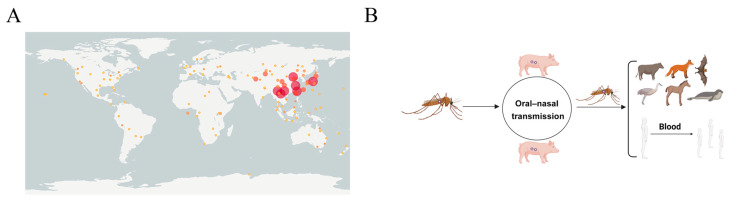
Vector and transmission cycle of JEV. (**A**) Global geographical distribution of *Culex tritaeniorhynchus*. The dots in the figure represent the distribution of *Culex tritaeniorhynchus*, with larger and redder dots representing a broader and more abundant distribution (**B**). Transmission of JEV. Mosquitoes were the primary vector, and pigs were the amplifying host. Subsequently, mosquitoes transmitted JEV through a bite.

To further explore the impact of vector species on JEV transmission, a distribution map of *Culex tritaeniorhynchus* was obtained from the GBIF website. It was revealed that *Culex tritaeniorhynchus* was mainly distributed in Asia, especially in China and Japan (Figure 3A). These countries had a high number of JEV sequences uploaded and a high disease prevalence. The major distribution of *Culex tritaeniorhynchus* was consistent with the collection of whole-genome sequences. The international spread of JEV has been facilitated by the migratory life of this mosquito species. America, which was geographically isolated from Asia by oceans and thus faced geographic constraints, had 0 JEV whole-genome sequences. Therefore, it was one of the good ways to combat JE through controlling *Culex tritaeniorhynchus* in these countries. Besides *Culex tritaeniorhynchus*, other mosquito species, such as *Anopheles sinensis* and *Armigeres subalbatus,* were also involved in JEV transmission based on whole-genome sequence information. It is well known that JE occurs mainly in areas characterized by rice cultivation and flood irrigation practices, which tend to breed Culex mosquitoes and attract wading birds. Mosquito species serving as vectors are prone to biting people. Furthermore, the rapid development of agriculture, large populations, and active pork trade across various countries heightened the risk of JEV infection while facilitating its spread, resulting in a high incidence of JEV in this region [73]. This was consistent with the results of our study, which indicated that the concentrations of *Culex tritaeniorhynchus* were highest in the country with the most JEV uploaded sequences and the highest incidence of JE. The mosquito–pig–mosquito cycle was important for the amplification and dissemination of JEV. JEV was amplified in pigs and spread through their mouths and noses in pig herds [74]. After mosquitoes bit infected pigs, JEV was carried and disseminated to other hosts, such as humans and horses, through biting. And JEV could spread in a population through the bloodstream (Figure 3B). In terms of the transmission cycle, controlling the amplification of JEV in pigs may be one of the effective ways to control JEV epidemics. By clarifying the transmission cycle, we can provide a reference for JEV to carry out integrated control and prevention.

### 3.3. Differences in Different Epitopes and Amino Acid Variants of E and NS1 Proteins

**Figure 4:**
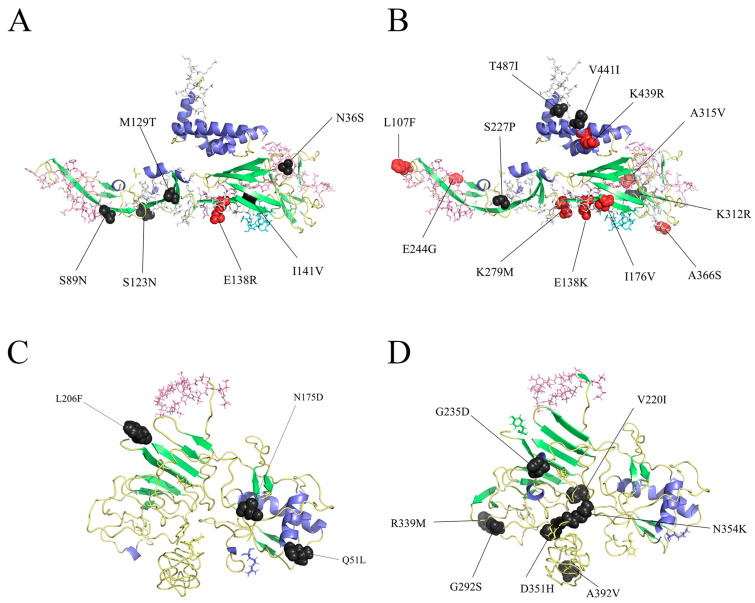
Structural prediction, epitope information, and amino acid variants of the E and NS1 proteins of GI and GIII. (**A**) E protein structure of JEV GI. (**B**) E protein structure of JEV GⅢ. (**C**) NS1 protein structure of JEV GI. (**D**) NS1 protein structure of JEV GⅢ. Protein structure models are colored according to their secondary structures: lime for the sheet, pale yellow for the helix, and slate for the loop. The red spheres represent mutation sites that have been shown to affect JEV function through reverse genetic experiments. The dark gray spheres indicate the amino acid variants with high mutation frequencies counted in this study. And the ball-and-stick shapes are the divergent amino acids of the GI and GIII amino acid coding sequences used in the modeling. The white line shapes are T-cell epitopes, while the pink lines are T-cell epitopes. The aquamarine lines are the overlap between the T-cell and B-cell epitopes.

Structural protein E was related to entry, assembly, and release and affected viral virulence. Nonstructural protein NS1 was functionally critical for viral immune evasion. Therefore, we focused on analyzing the amino acid variants of these proteins. The analysis of the whole-genome sequences of the JEV GI and GIII showed 62 mutation sites and 76 mutations in the E protein of the GI strains (Figure 4A; Table S2). The E protein of the GIII strains had 66 mutation sites and 73 mutations (Figure 4B; Table S2). There were 38 mutation sites and 40 mutations in the NS1 protein of the GI strains (Figure 4C; Table S2). There were 33 mutation sites and 36 mutations in the NS1 protein of the GIII strains (Figure 4D; Table S2). Some mutation sites were mutated to different amino acids. The amino acid coding sequences of the E genes from the GI and GIII strains exhibited four differences, of which site 366 was the site with a high frequency of amino acid mutations in this study. Meanwhile, the amino acid coding sequence of the NS1 gene from GI and GIII was shown to have eight amino acid differences, with higher frequency mutations at sites 51, 175, and 206. These variations may have different effects on viral functionality. SWISS-MODEL and I-TASSER were used to predict the protein structure of the E and NS1 proteins of the JEV GI strains and JEV GIII strains, respectively. The major mutation sites were labeled in Figure 4. Among these, reverse genetic studies on the L107F, E138K, I176V, E244G, K279M, A315V, S366A, and K439R mutations in the JEV E protein were reported [75,76,77,78,79,80]. In addition, the presence of mutations such as S89N had a high mutation frequency, which warrants further in-depth exploration of their functional impacts. The mutations of Q51L, N175D, L206F, V220I, G235D, G292S, R339M, and D351H in the JEV NS1 protein had an upper frequency. However, there was a relative scarcity of studies focusing on the NS1 protein. In this study, we provided several higher frequency mutations to enhance our understanding of genetic variation concerning the structure and function of both JEV E and NS1. For the E protein, ten identical mutations were found between GI and GIII, while only three were observed for NS1. Interestingly, among these mutations, the S123N mutation in the E gene had a significantly higher frequency in GI compared to GIII. This change is mainly due to a mutation at position 2 of the codon encoding this amino acid, resulting in a mutation from G to A at position 368 of the E gene nucleotide. Such alterations may have influenced the evolutionary differences between GI and GIII. According to the known epitope information documented in the IEDB database for JEV, the E protein of GIII has one more segment of T epitope information for VGRLVTVNPFVATSSANSKV compared to GI, while both exhibit identical B-cell epitope information. The NS1 protein lacked documented T-cell epitope information and had only one linear B-cell epitope. Linking amino acid mutations to epitope regions revealed that 22% of the kinds of amino acid mutations in the E protein of GI were located in epitope regions, whereas GIII exhibited a higher percentage at 31.5%. For example, T487I was present only in GIII and was located in the T-cell epitope region. Meanwhile, the NS1 protein of GI had a greater number of amino acid mutations within the epitope region than GIII (Figure 4; Table S3). This observation indicated that immune selection pressure promotes the evolution of JEV. Whether the amino acid mutations will influence the epitope or antigenic effect remains to be validated through further experiments.

### 3.4. Predicted Differences in Glycosylation and Palmitoylation Sites Across Genotypes

**Figure 5:**
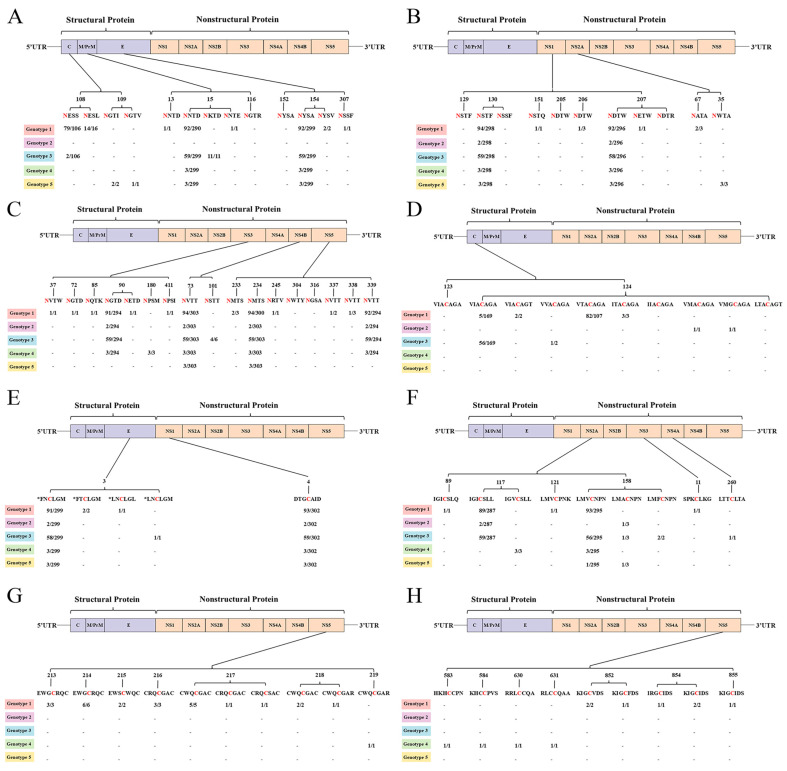
Prediction of functional sites in the JEV protein. (**A**–**C**) Glycosylation sites of JEV protein. Potential N-Glycosylated asparagine is marked in red. (**D**–**H**) Palmitoylation sites of JEV protein. Palmitoylation sites are highlighted in red. The first row of numbers under the genome structure represents the predicted functional sites. “-” represents no signal detected.

Protein post-translational modifications, such as phosphorylation, acetylation, ubiquitination, and SUMO modification, can widely participate in the regulatory process of viral induction of type I interferon production, and viruses can use the post-translational modifications of proteins to remove their adverse factors and escape the attack of the host immune system [81]. Therefore, to further investigate the impact of functional differences in post-translational modifications of proteins from different genotypes on viruses, amino acid sequences of JEV C, M/PrM, E, NS1, NS2A, NS2B, NS3, NS4A, NS4B, and NS5 proteins were intercepted. This was conducted for the prediction of SUMOylation sites as well as N-glycosylation and palmitoylation functions. The results showed that none of the JEV proteins contained SUMO sites. 32 N-glycosylation sites and 17 palmitoylation sites were predicted. The data statistics showed that the NS4A amino acid sequences lacked the N-glycosylation site, and the M/PrM and NS4B amino acid sequences lacked the palmitoylation functional site. In contrast, the nonstructural protein NS2B contained neither N-glycosylation nor palmitoylation functional sites (Figure 5). The glycosylation sites at positions 130 and 207 of NS1, 73 of NS4B, and 234 of NS5 were common to all five genotypes. In contrast, the glycosylation sites at amino acid residues 154 of the E protein were absent only in GII. Previous studies have shown that the N-glycosylation site N154 of the E protein has a significant effect on humoral immunity to JEV. Our study indicated that this glycosylation site was retained in essentially all genotypes. The lack of this site in GII may be due to the presence of specific variants. The prediction of glycosylation sites in E proteins will contribute to further understanding JEV pathogenesis and vaccine development. Regarding palmitoylation, position 3 of the E protein and position 4 of the NS1 were found in all the genotypes. The different N-glycosylation sites and palmitoylation sites presented among different genotypes of JEV may influence their function. Additionally, amino acid variability at functional sites may further affect the virulence and antigenic effects of JEV.

### 3.5. The Evolution Factor of JEV

Phylogenetic analysis of JEV implied that its evolution may be influenced by both geographical and host-related factors. To explore the evolutionary relationships between different JEV strains, whole-genome sequences with detailed source, genotype, and host information were constructed as a phylogenetic tree. The information contained was shown on the evolutionary tree, where the inner circle was the genotype information. Consistent with previous studies, all JEV whole genomes were categorized by five genotypes. The majority of these sequences corresponded to GI and GIII, with China identified as the primary country.

**Figure 6:**
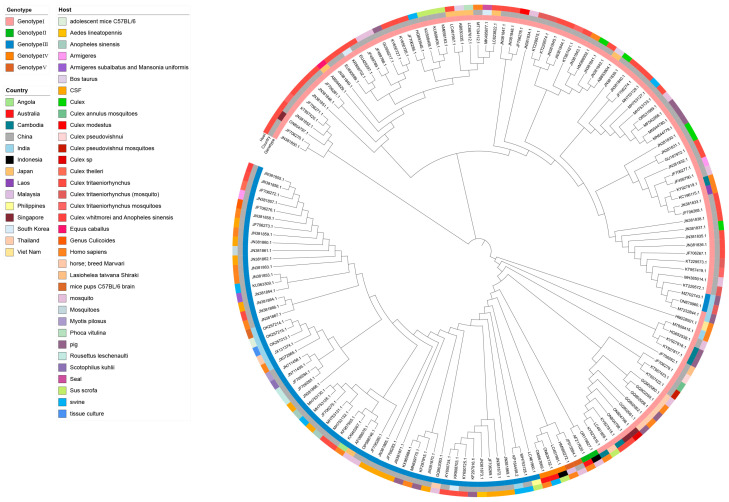
Phylogenetic tree of the whole genome of JEV. The outer circle of the phylogenetic tree was the host information of the JEV whole-genome sequences, the middle was the country of origin, and the inner circle was the genotype information.

JEV GII strains were found to be adjacent to GI strains, while GIV strains were closely related to JEV GIII strains. GV strains were in the middle of GII and GIV. Among them, MZ702743.1 and ON875960.1, which belonged to GIII, were positioned between GI strains KT229572.1 and MT232844.1. This positioning may be related to their geographical location and host (Figure 6). MZ702743.1, ON875960.1, and MT232844.1 are all from India. Although ON875960.1 and KT229572.1 were not in the same country, they were geographically proximate and shared a common host, *Culex tritaeniorhynchus*, likely contributing to their evolutionary connection. All the genotypes have been isolated from mosquitoes except GIV, which has not been isolated from mosquitoes. It was probably because GIV contained too few whole genome sequences. JEV was co-transmitted among various animals. Furthermore, GII and GIV strains originated from Australia in Oceania and Indonesia in Asia, with isolated hosts including humans and mosquitoes, as well as humans and pigs, respectively. GV originated from China and Malaysia, with its hosts comprising humans and mosquitoes. Furthermore, phylogenetic relationships indicated that Chinese strains GI and GIII shared similar developmental relationships with strains from other Asian countries, both of which had a wide distribution of mosquitoes. Particularly, the migration of mosquito species as well as trade activities to and from China further contributed to the widespread dissemination and evolution of JEV.

### 3.6. The Characters of JEV Evolution

**Figure 7:**
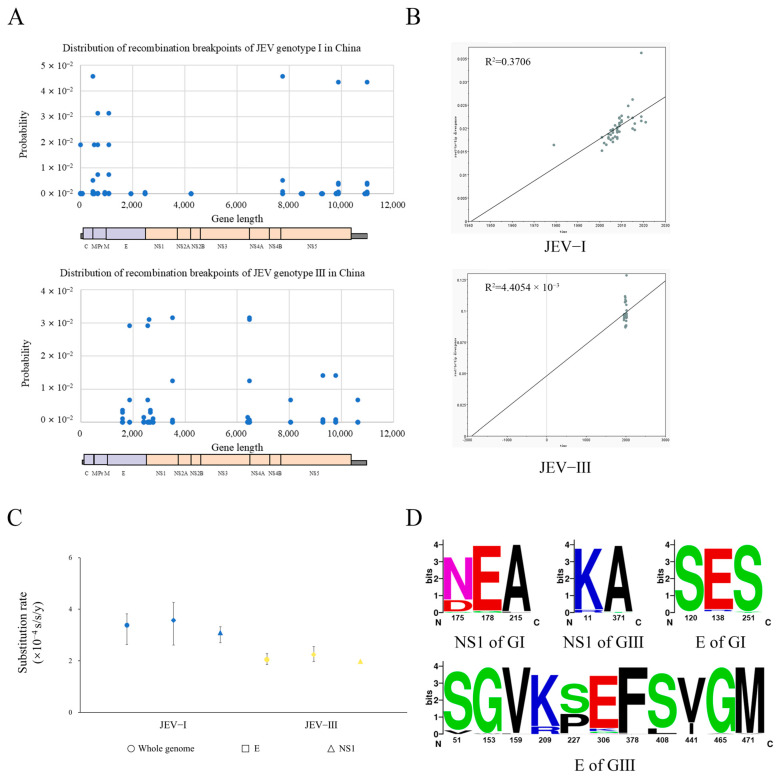
Gene recombination and the evolutionary rate of JEV. (**A**) Distribution of recombination breakpoints in the whole genome of JEV in China. With gene length as a horizontal coordinate and breakpoint distribution probability as the vertical coordinate, X and Y scatter plots were drawn. (**B**) Time signal diagram of GI and GIII based on root-to-tip distance analysis. The horizontal coordinate is time, and the vertical coordinate is root-to-tip distance. (**C**) Substitution rate sizes and comparisons for the whole genome, E gene, and NS1 gene. Blue represents JEV GI, and yellow represents JEV GIII. Orbicular represents the mean evolutionary rate of the whole genome, rhombic represents the E gene, and triangle represents the gene NS1. (**D**) Comparison of amino acid variation trends in the E and NS1 genes of GI and GIII at the episodic diversifying selection sites predicted based on the MEME algorithm. Each letter represents an amino acid. The graph represents the amino acid variation at the selective pressure sites; a larger letter indicates the letter with more sequences at that site.

To further explore the evolutionary characteristics of JEV, we analyzed the characterization of the distribution of JEV recombination breakpoints and compared evolutionary rates. Genetic recombination facilitates viral adaptive evolution to its environment [82]. All the sequences were analyzed for genetic recombination separately by country using RDP 4 software. The results showed that most countries had too few sequences for RDP 4 analysis, with some having 0 recombination events. Singapore and South Korea had two and three recombination events, respectively (Table S4). This suggested that genetic recombination occurs within a small geographic area. The country with the most recombination events was China, indicating that JEV in China has a faster rate of reorganization compared to those in other countries. Furthermore, all the Chinese JEV sequences were analyzed by genotypes. The results showed 10 recombination events for the Chinese GI strains and 8 for the GIII strain. Other genotypes were excluded from gene recombination analysis due to insufficient sequence data. The scatter plot showed that recombination events for Chinese GI strains occurred in the JEV C, M/PrM, E, NS2A, NS4B, and NS5 proteins, whereas recombination events for the GIII strains occurred in the E, NS1, NS3, NS4A and NS5 proteins (Figure 7A; Table S5). Specifically, the recombination breakpoints in GI were mainly distributed between the C, M, and E genes, whereas GIII was mainly distributed within the E gene and at the E-NS1 boundary. These differences in recombination breakpoints between GI and GIII may explain their evolutionary divergence.

Sequences excluding recombinant sequences with known information on time, country, genotype, and isolation location were analyzed. Consequently, 83 alignments were retained, comprising 46 GI, 2 GII, 31 GIII, 2 GIV, and 2 GV sequences. Root-to-tip distance analysis showed JEV genotype I (JEV-I) and JEV genotype III (JEV-III) had a time signal, though the R^2^ was small (Figure 7B). Further, the results of the mean evolutionary rates of JEV-I and JEV-III were comparable. For the whole genome, 3.3830 × 10^−4^ and 2.0481 × 10^−4^ substitutions/site/year were for JEV-I and JEV-III. Comparative analyses of the evolutionary rates revealed that JEV-I evolved more rapidly than JEV-III on the whole-genome level. For the E gene, the mean evolutionary rates of JEV-I and JEV-III were 3.5650 × 10^−4^ and 2.2423 × 10^−4^, respectively. The evolutionary rate obtained for JEV-I was higher than that of JEV-III, which was the same as that of the whole genome. However, the evolutionary rates of the E gene were higher than those of the whole genome. For the NS1 gene, the evolutionary rates obtained for JEV-I were about 56.11% higher than those of JEV-III, which may be related to selection pressure (Figure 7C; Table S6). Interestingly, the evolutionary rate of the JEV-I NS1 gene was about 8.83% lower than that of the whole genome, while the JEV-III NS1 gene was about 3.53% lower. This finding was consistent with the fact that GI has evolved more rapidly than GIII in recent years and therefore explained the gradual emergence of GI as the dominant genotype. Using a single-factor analysis of variance on SPSS 26, the evolutionary rates of the whole-genome sequences, E gene, and NS1 gene from the GI and GIII sequences showed no significant difference. It implied that the whole-genome sequences, E gene, and NS1 gene for JEV were consistent.

A further MEME analysis revealed that the E gene of GI exhibited three sites under episodic diversifying selection, whereas GIII had eleven such sites. In terms of the NS1 gene, GI had three diversifying sites, while GIII contained two. An analysis of the amino acid variants at the above selective pressure sites showed that the NS1 gene of JEV-I contained sequence variants, which may have driven the evolution of the NS1 gene in GI (Figure 7D; Table S7). Based on the relative GTR branch lengths and nucleotide substitution biases, the ratio of non-synonymous to synonymous rates for the E gene was 0.0578 for GI, while for GIII it was 0.0893. For the NS1 gene, the ratio of non-synonymous to synonymous mutations was 0.0540 for GI and 0.0579 for GIII. This suggested that both genes were affected by negative selection but also exhibited evidence of positive selection. As further corroborated by three algorithms, the E gene of GIII exhibited one site under positive selection pressure, specifically at position 227. In contrast, the NS1 gene of GI showed a site under selection pressure at position 175 (Table S8). According to the BUSTED analysis, except for the NS1 gene of GIII (*p* = 0.1617), evidence of episodic diversifying selection was observed in the NS1 gene of GI (*p* = 0.01765) as well as in both E genes of both GI (*p* = 0.0005249) and GIII (*p* = 1.682 × 10^−10^).

### 3.7. Phylogenetic Geographical Analysis of JEV

**Figure 8:**
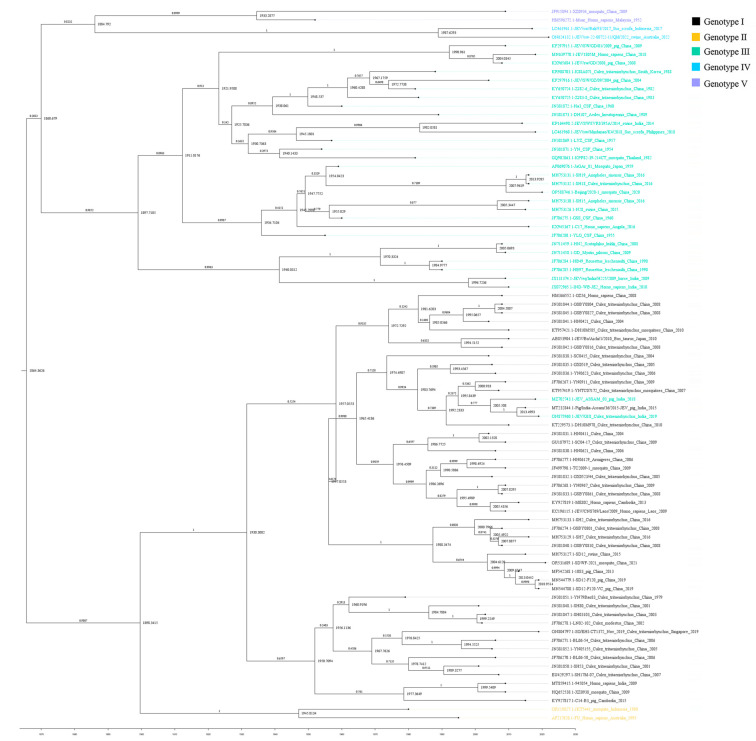
Maximum clade credibility tree of the JEV E gene. Sequence names were colored differently depending on the genotype. Node numbers represented time, and branch numbers represented posterior probability.

The sequences of gene E, with recombinant sequences removed from all whole-genome sequences of JEV and accompanied by information on time, country, genotype, and isolation location, were reconstructed for the Bayesian phylogenetic analysis. It revealed that the rooted tMRCA was 1864.3626 for the entire dataset with posterior probability (PP) = 1. In 83 sequences, the earliest JEV sequence was found in Malaysia in 1952, which was genotype Ⅴ. GII had an estimated tMRCA of 1945.8134, with PP = 1. And the tMRCA of GIII was 1897.7105, with PP = 0.9852. However, MZ702743.1 and ON875960.1, which belonged to GIII, were phylogenetically related to GI. It was probably because they all took place in India and at a similar time with the same clade. The tMRCA of GI, which was obtained above the two sequences, was 1930.3002, with PP = 1. GI was isolated from *Culex tritaeniorhynchus* in 1979 in China. The tMRCA of GIV and GV was 1987.6293, with PP = 1, and 1933.2877, with PP = 0.9999, respectively. It was found that the evolutionary order was GIII, GI, GV, GII, and GIV. Overall, GIII, GIV, and GV had a closer phylogenetic relationship, while GI and GII were closer. This was consistent with the results of the ML tree (Figure 8).

## 4. Discussion

Since the first report of JEV in Japan in 1871 [83], its circulation has extended to China, India, and various countries in Southeast Asia [84,85]. However, with the acceleration of world trade circulation, the epidemic area of JE is no longer limited to Asia and gradually expanded to the Pacific region and even Oceania [86]. In this study, we found that the whole-genome sequences of JEV were predominantly collected from Asian countries, particularly China. The analysis of whole-genome sequence information indicated that the dominant epidemic strains in China are GI and GIII. Consequently, the focus of prevention and control efforts should be directed toward these two genotypes, which is consistent with previous studies [10]. China is significantly affected by JE, exhibiting a broad geographic distribution of the disease. Furthermore, the co-existence of multiple genotypes in certain provinces increases certain management difficulties, especially in Yunnan Province. Yunnan, which borders Southeast Asia, was a high-prevalence area for many arboviruses and served as a transportation hub, which further facilitated the cross-border introduction of arboviruses into Yunnan, increasing the risk of arboviral disease epidemics [87,88]. Moreover, Yunnan Province had a tropical–subtropical climate, with high average annual temperatures and abundant precipitation providing favorable conditions for arthropod reproduction, further increasing the risk of arbovirus transmission. Therefore, it is necessary to monitor and control JEV in various places in Yunnan Province. JEV host species were diverse, with most uploaded JEV sequences originating from mosquitoes. *Culex tritaeniorhynchus* mosquito surveillance should be carried out continuously to provide basic data for JE surveillance through the knowledge of its population density and habitat differences [89]. Our evolutionary analysis of the ML tree showed that JEV strains from most Asian countries were closely related and exhibited a wide distribution of mosquitoes. Based on comprehensive whole-genome sequence information and the relevant literature, it can be concluded that JEV has spread to cattle and seals, posing an increased threat to public safety. Other hosts of JEV, such as bats and midges, play an important role in the long-distance transmission of JEV [90,91], showing that JEV has the potential for further transmission. Therefore, enhanced control measures at ports remain essential for effective quarantine management.

Previous studies suggest that amino acid variants in the E protein will have an impact on the virulence of JEV, with various amino acid mutations playing an important role in attenuated JEV strains. Through genetic evolutionary analysis of JEV E and NS1, it was found that numerous variation sites exist within the amino acids of both JEV E and NS1. Overall, the coding amino acids of both the E protein and the NS1 protein in GI had a greater diversity of amino acid mutations compared to those in GIII. Previous studies have shown that sites 107, 138, and 176 of the JEV E protein have important effects on the virulence of JEV [75,76,77], and mutations at site 244 of the E protein changed the infectivity of JEV [78]. And L107F, E138K, I176V, E244G, K279M, A315V, S366A, and K439R are responsible for the weakening of JEV neurovirulence [79]. The monitoring of amino acid residues and mutations of E proteins such as E107, E138, E123, E176, E177, E244, and E279 can provide a theoretical basis for the safety mechanism of a JEV attenuated vaccine and contribute to the development and quality control of new JEV vaccines [80,92]. In the study, amino acid mutations at the aforementioned key sites were observed, and differences in these sites between GI and GIII were obtained. The L107F, E138K, I176V, E244G, K279M, A315V, and K439R mutations that attenuate the neurovirulence of JEV were found only in GIII, and not in GI, which is consistent with the prevailing trend of GI gradually replacing GIII nowadays. There were a few reports about the reverse inheritance of JEV NS1. We analyzed the amino acid mutation of NS1 of JEV GI. The results showed that the N175D, L206F, and Q51L mutations had high frequencies. A comparative analysis of JEV GIII showed that six kinds of mutations, V220I, G235D, G292S, R339M, D351H, and N354K, occurred more frequently. However, the significance of the aforementioned JEV mutation sites was still unclear, and whether they affect the neurovirulence and infectivity of JEV needed to be further studied in conjunction with molecular dynamics studies. In addition to the high-frequency mutations listed above, we have prepared an exhaustive table of amino acid mutations in the E and NS1 proteins of GI and GIII. This table aims to provide a basis for epidemiological studies and vaccine development for targeting JEV.

In this study, we integrated the epitope information of the JEV E protein and NS1 protein recorded in the IEDB database with the amino acid mutation profile. This integration provides a valuable reference for future immunohistochemistry studies. Differences in T-cell epitopes within the 351–370 region of the E gene between GI and GIII may be attributed to amino acid mutations. Although the B-cell epitopes in both genotypes were identical in the E and NS1 proteins, the amino acid mutation situation within the epitopes was quite different. Interestingly, in addition to several amino acid mutations exhibiting high mutation frequencies, mutations that affect the epitopes of the E structural proteins were also observed, including L107F, C60Y, and N103K. The L107F mutation is located within the FL region, which is responsible for the membrane fusion of viruses. The mutant VLP was found to be completely unresponsive to 3H12. It has also been shown that the L107 residue is a part of the 3H12 epitope. The levels of anti-JEV antibodies induced by these mutant vaccines are comparable to wild-type DNA vaccines [93,94]. In addition, mutants C60Y and N103K in domain II were predicted to be B-cell epitopes and T-cell populations. Studies have shown that these mutations affect the stability of the E protein, leading to patients infected with these mutated strains escaping neutralization by a GIII-specific vaccine [95]. However, fewer studies are focusing on mutations in the epitope of the NS1 gene. This study, however, provides the amino acid mutations present in the B-cell epitopes of the coding amino acid sequence of the NS1 gene. The GI has a greater variety of amino acid mutations at the DTGCAIDITRKEMRC epitope, despite having a low frequency of amino acid mutations at this epitope. Consequently, the effects of mutations on epitopes and immunity can be further analyzed. The mutation analysis of JEV presented in this study offers valuable insights into the vaccine development for JEV.

Glycosylation has affected the spatial conformation, activity, transport, and localization of proteins and played a crucial role in signal transduction, molecular recognition, immunity, virus replication, infection, and virulence [92,96,97,98]. Previous studies have found that the loss of N-glycosylation at prM site 15 resulted in an approximately 20-fold reduction in the production of extracellular virions, which have almost the same protein composition and infectivity as wild-type virions [99]. In this study, the prediction of glycosylation sites for individual JEV proteins revealed that only GII is deficient in N-glycosylation at the prM site 15. This deficiency may be one of the reasons why the GII genotype is less prevalent than the other genotypes and has led to the fact that fewer whole-genome sequences have been uploaded on the subject. At the same time, glycosylation was found at the N154 site of the E protein in all the genotypes except for GII. The glycosylation site of the JEV E protein was critical for viral replication, infectivity, pathogenicity, and neurotoxicity [98], and the mutation of the N-glycosylation site N154 of the E protein significantly enhanced the virus-induced humoral immune response, and E protein glycosylation regulated the release of subviral particles [100,101]. The reason for the deletion of this site may be the presence of a specific mutation in GII. Palmitoylation played a crucial role in host–virus interactions [102]. However, there were fewer JEV palmitoylation studies available. In this study, we predicted the functional sites of each JEV protein, which can provide reference data for the relevant research on post-translational modification targeting JEV.

Genetic recombination promotes adaptive evolution of viruses. However, there are fewer studies on recombination in the JEV whole genome [82]. In this study, we analyzed the recombination of the JEV whole genome across different countries through RDP 4. It was found that no recombination events were detected except for those in China, Singapore, and South Korea. This is consistent with the results of previous studies that found that recombination in JEV occurs on a relatively smaller scale [103]. In addition to this, we further explored the evidence of recombination of GI and GIII in Chinese JEV strains. It was found that the GIII genotype recombination occurred within the E gene. This may affect both the infectious ability and virulence of the GIII.

The rate of molecular evolution can be used to evaluate the evolutionary characteristics of viruses. In this study, we found that JEV-I mutated faster than JEV-III from the whole genome, as well as in both the E gene and the NS1 gene, by calculating the substitution rate of nucleotides in a given period. This is consistent with the gradual replacement of JEV-III by JEV-I as the prevalent strain, suggesting that these genetic substitutions have facilitated the adaptive evolution of JEV. However, we were missing the calculation of the evolutionary rates of other genotypes. This is because our data came from sequences with the recombination removed. Other genotypes had too few sequences to allow for evolutionary analysis. The high mutation rate of nucleotides was one of the important factors leading to the strong adaptability and high pathogenicity of viruses [104]. The MCC-reconstructed tree based on E of JEV revealed the evolutionary order of JEV genotypes. By analyzing the tMRCA, we found that the evolutionary order was GIII, GI, GV, GII, and GIV. The result was different from other research, which showed the order was GV, GIII, GII, GI, and GIV [104]. This was probably because we used sequences with the recombination removed and used the E gene rather than the whole genome. E gene, as a key gene of typing, was associated with virus evolution. We can rapidly analyze the evolution of JEV by analyzing the E gene. However, due to technology, we have not been able to conduct a spatiotemporal geographical analysis of JEV. Our ESS for the selected parameters of the MCC tree exceeded 100. According to the BEAST website, we do not need adequate ESSs for all parameters. But it may have implications for the tree regarding an estimate of the posterior distribution.

So far, there has been no specific clinical drug that can be used to treat JE; one can only choose to take symptomatic therapy and supportive therapy. Therefore, the prevention of JE was particularly critical. In this study, we conducted a comprehensive analysis of the whole-genome sequence of JEV using bioinformatics software to clarify the epidemiological dynamics, phylogenetic analyses, and protein structural differences. We investigated recombination events and evolutionary rate differences between the two genotypes, GI and GIII, specifically focusing on the E and NS1 genes. This study demonstrated the evolutionary dynamics of JEV and provides a reference for future in-depth studies related to vaccines and genomics research.

However, we have only included the whole-genome sequence of JEV that has been uploaded to the NCBI database in this study, and it lacked the comprehensive epidemiological data on cases of viral infection, infected animals, and seroprevalence. The effects of evolution on viral structure and function have not been explored in depth. Therefore, this study was only a preliminary exploration; a comprehensive exploration requires linking clinical and epidemiologic data, which will be further improved in the future.

## Data Availability

The original contributions presented in this study are included in this article/Supplementary Material. Further inquiries can be directed to the corresponding authors.

## Supplementary Materials

The following supporting information can be downloaded at https://www.mdpi.com/article/10.3390/microorganisms13061226/s1: Table S1: Details of 303 whole-genome sequences; Table S2: Gene E and NS1 amino acid mutation of JEV; Table S3: T- and B-cell epitope information for E and NS1; Table S4: Table of recombination events by country; Table S5: Recombination events of Chinese JEV; Table S6: Evolutionary rate of JEV with recombinant sequences removed; Table S7: Amino acid composition at sites affected by episodic diversifying selection as detected by Mixed-Effects Model of Evolution (MEME); Table S8: Prediction of positively selected sites for the E and NS1 genes.
